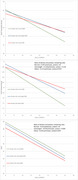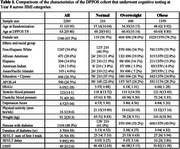# Association of Body Mass Index and Cognitive Performance in the Diabetes Prevention Program Outcomes Study

**DOI:** 10.1002/alz70860_098699

**Published:** 2025-12-23

**Authors:** Jose A. Luchsinger, Qing Pan, William C Knowler, Medha Munshi, Karol Watson, Kishore M Gadde, Mathias Schlögl, Owen T. Carmichael

**Affiliations:** ^1^ Columbia University Irving Medical Center, New York, NY, USA; ^2^ George Washington University, Washington, DC, USA; ^3^ George Washington University, Rockville, MD, USA; ^4^ Joslin Diabetes Center, Boston, MA, USA; ^5^ UCLA David Geffen School of Medicine, Los Angeles, CA, USA; ^6^ University of California Irvine (UCI) School of Medicine, Irvine, CA, USA; ^7^ Clinic Barmelweid, Erlinsbach, Erlinsbach, Switzerland; ^8^ Pennington Biomedical Research Center, Baton Rouge, LA, USA

## Abstract

**Background:**

Persons with prediabetes or type 2 diabetes (T2D), representing two thirds of the adult US population, are at increased risk of cognitive impairment. A better understanding of how excess adiposity (typically quantified by the body mass index, BMI) influences cognitive function in the specific context of prediabetes or T2D could help to inform strategies for prevention of cognitive decline during long‐term treatment of individuals with prediabetes or T2D.

**Method:**

We completed a repeated measures analysis of data from the Diabetes Prevention Program Outcomes Study (DPPOS). We used BMI as a surrogate of adiposity, categorized as normal (< 25 k/m^2^), overweight (25 to <30 k/m^2^), or obese (^3^ 30 k/m^2^). Cognitive tests included the Spanish English Verbal Learning Test (SEVLT) immediate recall and delayed recall, and the Digit Symbol Substitution test (DSST). The relationship between BMI at the DPPOS year 8 visit, and cognitive test scores at DPPOS years 8, 10 and 15 visits, was ascertained jointly via linear mixed models accounting for repeated measures. Analogous models assessed the relationship between BMI at DPPOS year 15, and the modified mini mental exam (3MS) score at that same time point.

**Result:**

We completed a repeated measures analysis of data from the Diabetes Prevention Program Outcomes Study (DPPOS). We used BMI as a surrogate of adiposity, categorized as normal (< 25 k/m^2^), overweight (25 to <30 k/m^2^), or obese (^3^ 30 k/m^2^). Cognitive tests included the Spanish English Verbal Learning Test (SEVLT) immediate recall and delayed recall, and the Digit Symbol Substitution test (DSST). The relationship between BMI at the DPPOS year 8 visit, and cognitive test scores at DPPOS years 8, 10 and 15 visits, was ascertained jointly via linear mixed models accounting for repeated measures. Analogous models assessed the relationship between BMI at DPPOS year 15, and the modified mini mental exam (3MS) score at that same time point.

**Conclusion:**

Among persons with prediabetes or T2D in DPPOS, overweight or obesity was associated with slower decline in verbal memory performance compared with those with normal BMI. The causality and mechanisms for this association need to be determined in future studies.